# The effect of financial incentives on chlamydia testing rates: Evidence from a randomized experiment^☆^

**DOI:** 10.1016/j.socscimed.2013.11.018

**Published:** 2014-03

**Authors:** Paul Dolan, Caroline Rudisill

**Affiliations:** Department of Social Policy, London School of Economics & Political Science, Houghton St., London WC2A 2AE, UK

**Keywords:** UK, Financial incentives, Sexually transmitted infections, Socioeconomic status

## Abstract

Financial incentives have been used in a variety of settings to motivate behaviors that might not otherwise be undertaken. They have been highlighted as particularly useful in settings that require a single behavior, such as appointment attendance or vaccination. They also have differential effects based on socioeconomic status in some applications (e.g. smoking). To further investigate these claims, we tested the effect of providing different types of non-cash financial incentives on the return rates of chlamydia specimen samples amongst 16–24 year-olds in England. In 2011 and 2012, we ran a two-stage randomized experiment involving 2988 young people (1489 in Round 1 and 1499 in Round 2) who requested a chlamydia screening kit from Freetest.me, an online and text screening service run by Preventx Limited. Participants were randomized to control, or one of five types of financial incentives in Round 1 or one of four financial incentives in Round 2. We tested the effect of five types of incentives on specimen sample return; reward vouchers of differing values, charity donation, participation in a lottery, choices between a lottery and a voucher and including vouchers of differing values in the test kit prior to specimen return. Financial incentives of any type, did not make a significant difference in the likelihood of specimen return. The more deprived individuals were, as calculated using Index of Multiple Deprivation (IMD), the less likely they were to return a sample. The extent to which incentive structures influenced sample return was not moderated by IMD score. Non-cash financial incentives for chlamydia testing do not seem to affect the specimen return rate in a chlamydia screening program where test kits are requested online, mailed to requestors and returned by mail. They also do not appear more or less effective in influencing test return depending on deprivation level.

## Highlights

• This chlamydia testing study is one of the largest, most thorough incentives trials.
• Non-cash financial incentives had no impact on chlamydia testing for young adults.
• Incentives were no more or less effective depending on socioeconomic status.
• The results are surprising given the theoretical underpinnings of the incentives' designs.
• Context is important in the success of any policies designed to change behavior.

## Introduction

Financial incentives present policy options to change patient behavior in a number of areas including smoking and weight loss (Marteau, Ashcroft, & Oliver, 2009). Several reviews have concluded that financial incentives are successful in influencing ‘one-shot’ behaviors, such as immunizations and appointment attendance (Kane, Johnson, Town, & Butler, 2004; Sutherland, Christianson, & Leatherman, 2008). This study considers the generalizability of this conclusion by using a large, randomized experiment occurring in a natural setting to test the effectiveness of financial incentives in promoting chlamydia testing.

While effectiveness is one part of the decision to implement financial incentives, acceptability is another. A series of experiments examining the acceptability of financial incentives for smoking cessation and weight loss, found that the UK general public's acceptability of financial incentives increased with their level of effectiveness (Promberger, Dolan, & Marteau, 2012). Effectiveness is a crucial aspect to any successful incentive program in the eyes of the public, and so it is important to investigate whether and which types of incentives work most successfully.

In addition to effectiveness, considerations of equity also matter. A meta-analysis of trials found socioeconomic status to have an influence on the effectiveness of financial incentives applied in smoking, diet and physical activity contexts (Mantzari et al., in preparation). Policymakers could also use financial incentives to reduce health inequalities by targeting behaviors disproportionately engaged in by poorer people (Oliver & Brown, 2012). Incentives can be seen as coercive, however, even subtly forcing individuals to act in a way they do not wish, especially the more disadvantaged (Ashcroft, 2011; Lunze & Paasche-Orlow, 2013).

Beyond effectiveness and equity, considerations of financial sustainability are important for policy planning. Offering financial incentives presents a cost to health system payers. This immediate cost may or may not be worth the future costs avoided. The degree to which payers avoid future costs depends on incentive size and effectiveness (Giuffrida & Torgeson, 1997) as well as whether the effect of the incentive is sustainable over time (e.g. Volpp et al., 2008).

Chlamydia is the most common sexually transmitted infection (STI) in the United Kingdom (UK). The UK Department of Health's goal for 2010/11 was screening 35% of 16–24 year olds through the National Chlamydia Screening Programme (NCSP). The average across England was 28.5% from April 2011 to March 2012, ranging across English regions (Strategic Health Authorities) from 24.5% to 35.7% (NCSP, 2012a).

Young people return about 70% of chlaymdia test kits requested via Freetest.me, an online and text screening service run by Preventx Limited. 47 out of the 152 primary care trusts (PCTs)^1^ in England contract Preventx Limited to dispatch at-home chlamydia test kits requested by text or online as part of the NCSP. While 70% is an impressive return rate, it leaves almost 1/3 of dispensed tests unused. Therefore, we consider whether non-cash financial incentives might increase sample return rates and whether in a differential manner depending on socioeconomic status.

A number of studies have investigated the effect of financial incentives to encourage chlamydia screening but only a few have examined the use of incentives for mail-in chlamydia screening while including a control group (Molinar & Nardone, 2010). Low et al. (2007) found that offering a £10 voucher had no effect (compared to no incentive) on chlamydia screening uptake in a mail-based home screening program in England (*n* = 838). Niza, Rudisill, and Dolan (2013) tested the effect of offering a voucher (£5) and lottery participation (£200) on young adults' participation in chlamydia testing in four London student halls of residence (*n* = 1060). Incentives of any type were associated with a higher likelihood of participating in screening than those offered no incentive while the group offered a £5 voucher were more likely to return the test kit than those offered lottery participation. Zenner et al. (2012) compared areas of England that have employed patient financial incentives of any type and those that have not and found a small positive effect of offering vouchers but no effect of prize draws.

Currie et al. (2010) compared chlamydia screening participation in Australia when offering either education sessions and non-financial incentives over six months (*n*-2786) or four days of text messages and offering a cash incentive of AUD $10 (*n* = 866). The shorter text message/financial incentive strategy had a higher test uptake rate. Downing et al. (2012) found that offering a cash incentive of AUD $10 alongside of a text message reminder increased the likelihood that individuals who had previously tested positive for chlamydia would be re-tested in the recommended 3–4 month timeframe, but re-testing rates were still lower than desired (*n* = 94).

Against this background, financial incentives could be effective in increasing specimen return rates. Effectiveness alone should not determine the use of financial incentives – there are other ethical concerns (e.g. coercion, acceptability) and possible gaming effects that need to be considered – but evidence of effectiveness would, at least, suggest that these additional issues require closer scrutiny. Chlaymdia screening rates from the NCSP are highest in the most deprived parts of England where the populations are also at greatest risk of infection (Sheringham et al., 2011). Therefore, offering incentives could make differences in screening coverage across deprivation levels grow even wider. This study examines the effect of offering English 16–24 year olds non-cash financial incentives on their likelihood of returning a specimen sample for chlamydia testing. We also investigate the extent to which socioeconomic status influences incentive effectiveness.

Well-known theoretical concepts from behavioral economics that would be expected to affect behavior motivated the design of financial incentive schemes used in this study. We include lotteries because of evidence that people overweight low probabilities of high rewards (Loewenstein, Weber, Hsee, & Welch, 2001). Lotteries have also proved successful within contexts of financial incentives for health behaviors (Volpp et al., 2008). We also tested the choice of engaging in a lottery or receiving a certain reward of the expected value of a lottery option (both at the £5 and £10 levels) to examine whether allowing the choice between rewards might engage participants more deeply in decision-making regarding incentives as people prefer having options (Kamenica, 2012). The notion behind endowments is that by offering a participant a gesture of good-will or thanks, it might motivate reciprocity through kit return (Cialdini, 2001). Finally, the ability to give to charity taps into other-regarding motivations for behavior change (Burger & Lynham, 2010).

## Methods

### Setting and participants

We ran this study in conjunction with Preventx Limited's online and text screening service, Freetest.me. Individuals requesting test kits received them via post along with a pre-addressed stamped return box for their specimen sample (either urine or vaginal swab) to be sent to Freetest.me's laboratory in England for processing. The sample can be mailed back using a regular mailbox. Freetest.me notifies individuals that their results are ready via the method chosen (text or email) when requesting their test kit. They can retrieve results through an online tracking system on the Freetest.me website. Individuals can also request that they be called if the result is positive.

Internet and mobile (remote testing) are not the only way young people can take a chlamydia test in England. The biggest non-NCSP portion of chlamydia testing for young people between April 2011 and May 2012 was performed via genitourinary medicine (GUM) clinics, making up 27.5% of all tests across England. 54.2% of all tests were through the NCSP of which remote testing is part (4.4% of total chlamydia tests) as are GP-based tests (9.2%) (NCSP, 2012b). The breakdown of remote testing versus GP-based tests as well as other NCSP testing channels varies depending on the PCT. At the time of this study, Freetest.me tested about 50,000–60,000 patients annually.

Our study sample came from individuals requesting test kits through the Freetest.me Internet site (both via computer and mobile phone web access) and text message systems. It includes young people from all parts of England except those patients covered by the North East Strategic Health Authority (one of ten regional health bodies) because no PCT in this area contracted chlamydia screening through Preventx Limited at the time of this study.

Each test kit request was randomly allocated to an incentive or the control group (no incentive) sequentially as it came into Freetest.me. A slip (Appendices 1 and 2) was included in each kit with details of the randomly assigned incentive scheme. Freetest.me used a scanned barcode on each slip and a unique barcode identifier on the test kit itself as well as the specimen vial to keep track of participant randomization across control and treatment groups. To ensure patient confidentiality, we did not have access to the respondents' postcodes, which were needed to provide incentive rewards. Therefore, Preventx administered the incentives.

The research was funded by a Strategic Award from the Wellcome Trust Biomedical Ethics Programme. The funder had no involvement in this study's design, the collection, analysis and interpretation of data or the writing of the manuscript. This study received ethical approval from the London School of Economics & Political Science Research Ethics Committee.

This study involved two rounds (Rounds 1 and 2). Round 2 was undertaken to ensure the robustness of findings from Round 1. The first randomization process was from May 18–June 3, 2011. We had a target sample size of 1500 with test kit requests randomized across five incentive groups plus a control. We have data on 1489 kits as 11 were incorrectly scanned or labels fell off and therefore not traceable (0.7% lost).

The second randomization process was from May 8–18, 2012. Effect sizes from Round 1 informed power calculations for Round 2. We calculated the sample size needed for 80% power in showing the difference between groups and 5% two-sided error. Calculations were based on the return rate of the control group and the highest treatment group return rate in Round 1 and demonstrated a sample size of 300 for each group. To account for lost kits based on Round 1, we increased each group to 305. We had a target sample size of 1525 with requests randomized across four incentive groups plus a control. We have data on 1500 kits as 25 were not traceable (1.6% lost). We do not have Index of Multiple Deprivation (IMD) information for one participant in Round 2 therefore our sample is 1499 kits. This is total of 2988 across both Rounds. Appendices 3 and 4 show the flow of participants through request of text kit, intervention and follow-up in Rounds 1 and 2, respectively (Table 1).

We chose a Tesco voucher for each incentive group except the charity group because of the reward's universality. Tesco has stores across England and an online presence as a major English retailer. It is recognizable regardless of gender, socioeconomic status and interests. Tesco sells a wide range of products from food to clothing to electrical equipment. We chose ‘Children in Need’ as the charity because it is well-known across England and has a generally positive appeal. In both Rounds, respondents had 18 days to return their sample, after which they received the normal Freetest.me protocol of a text reminder. Freetest.me considers a kit invalid if individuals do not return the specimen sample 30 days from the request date. While specimen samples returned after 30 days are still tested by Preventx, for the purposes of this study, they are considered ‘non-returns’ rather than a ‘returns’.

### Data and statistical methods

Respondents could apply for their test kit either online or via text message. Those who requested online (through a computer or their mobile) had to fill out a questionnaire (Appendix 5) with personal details. We only have gender and postcode for those who requested via text. Those requesting a kit via Freetest.me's website supplied their gender, age, ethnicity, postcode, self-reported sexual history (whether respondent had new sexual partner in the last three months, more than two sexual partners in the past 12 months, used a condom the last time had sex and partner gender(s) in the last 3 months) and previous test history for chlamydia over the past 12 months including previous test results. We also know which PCT covers each participant, date of test request and date of test return (if applicable). We used respondents' PCTs to classify our sample by region (Department of Health, 2011).

Full postcodes determined each participant's IMD score (measure of socioeconomic status). The UK postcode system is very precise allowing for identification by actual street and not just area. IMD scores capture socioeconomic status through a weighted composite measure including seven domains (e.g. income, employment, health and disability) with 38 indicators in total (Department for Communities and Local Government, 2011). To maintain respondent anonymity, Preventx only supplied us with the first three letters of postcodes. Instead, Preventx sent respondent identification numbers and their full postcodes to a third party researcher. These researchers mapped postcodes supplied by Preventx to each participant's LSOA (Lower Super Output Area). Each participant's LSOA when then linked to the IMD score associated with his/her LSOA using the IMD 2010 index values.

We estimate the effect of any of the non-cash financial incentive structures on chlamydia test specimen sample return likelihood using multivariate logistic regressions clustering participants based on the first three letters of their postcodes. We use interaction terms to examine the extent to which participant socioeconomic status might influence the effectiveness of any of the incentives tested. We ensured specification robustness using variance inflation factors, the regression error misspecification test (Jones, 2007) and omitted variable bias checks. We performed all analyses in STATA 12.1.

## Results

### Study characteristics

The majority of respondents (95.5%) applied for their test kit online and thus had to fill out a questionnaire with personal details (*n* = 2850) to receive their test kit. Therefore, the response rate for the survey among this group was 100%. 4.6% of the sample (*n* = 138) requested via text therefore we only have their gender and postcode at a minimum. Table 2 displays descriptive statistics of our sample. It includes all respondents. Those requesting a kit by text and those who asked survey questions online but choosing the response ‘I'd rather not say’ were included as ‘unknown’ responses. Respondents were mostly white (86.5%), female (66.3%), had two or more sexual partners in the last twelve months (65.6%), had a new partner in the last three months (61.6%) and did not use a condom the last time they had sex (69.7%). The median age was 21 years. 41.1% of the group had taken a chlamydia test in the past year while 33.2% had already tested positive for chlamydia in the past year. The mean IMD score was 20.2 with a distribution ranging from 0.61 to 87.8. Our sample population is slightly less deprived than the general population of England, which has a mean IMD score of 21.7 (Pendle Council, 2010).

Baseline characteristics for all groups for both rounds were also similar (Tables 3 and 4). In order to elect the most statistically robust analysis plan, we tested whether the groups randomized to control and £5 voucher (the two arms common to both study rounds) were different from each other using ANOVA tests. If they were not, combining these two groups would be appropriate. The groups were not statistically significant different according to any characteristics except in two cases: for the control group, the percentage of Asian participants was lower in Round 2 (0.7%) than in Round 1 (3.2%) at a 5% significance level; and for the £5 voucher, the percentage of respondents having not used a condom the last time they had sex was higher in Round 1 (76.0%) than in Round 2 (65.7%) at a 1% significance level. Given similarities across treatment arms and the ability for our multivariate analysis to mediate some of these effects by controlling for study round, combining the samples allows us to examine the study with greater statistical power.

### Effect of incentives on specimen return rates

**Fig. 1:**
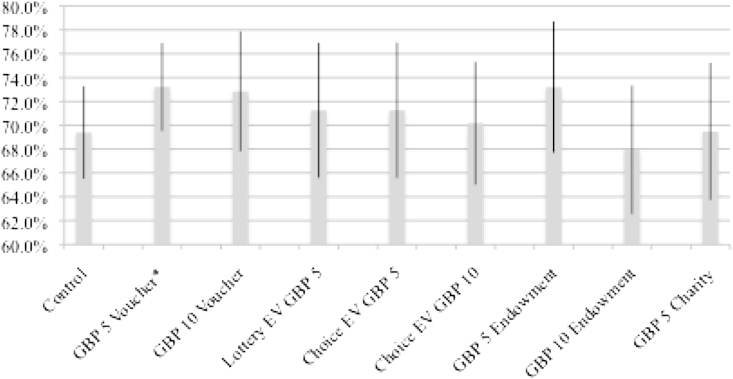
Percentage of test kits returned by incentive group. Note: Error bars represent 95% confidence intervals. *Difference between incentive and control group is significant at the 85% level.

The overall return rate was 71.0%. We see only very minor differences in return rates across incentive structures and none are statistically significantly different from the control or each other (Fig. 1 and Table 5). The group receiving the £5 voucher regardless of their behavior and those receiving the £5 voucher on sample return had the highest rates of return (73.2%) while those receiving an endowment of a £10 voucher had the lowest (67.9%). The control group had a return rate of 69.4%. For those given a choice of reward on returning their kit, the voucher was the more popular choice as 64.6% (56.8% when expect value (EV) was £5 and 71.5% when EV was £10) chose it versus only 11.3% electing to take part in the lottery (18.2% and 5.6%, respectively). When the value of the certain option (voucher) goes up, individuals are less willing to take the risk of the lottery. The remaining individuals in these treatment groups did not return a slip, did not fill it out or chose both options (24.1% total, 25.6% and 22.9%, respectively).

As for other factors influencing the likelihood of specimen sample return, those participants who were of lower socioeconomic status, younger (ages 15–19) were less likely to return a sample for testing while those who had previously tested positive for chlamydia were more likely to do so. Study round was not a significant predictor of sample return suggesting that any differential characteristics of the population samples in Rounds 1 and 2 did not introduce a bias.

### Socioeconomic status and incentives

While offering non-cash financial incentives appears to make no difference in return rates for the general population, they might matter for population sub-sets. We see that likelihood of returning a specimen sample was positively related to socioeconomic status. For a one unit increase in IMD score, the odds of returning a sample decreased by 1%. We used interaction terms to test whether IMD score would influence whether incentives make a difference in sample return. Table 6 displays the interaction between IMD and incentive structures demonstrating no differential effect of any of the incentives across deprivation level. Therefore, non-cash financial incentives worked similarly based on an individual's socioeconomic status.

## Discussion

In this randomized experiment, we showed that a variety of non-cash financial incentives made no difference to the rate of sample return in an online request, mail-back specimen chlamydia screening program targeting 16–24 year old English males and females. The sample size and randomized study design in a natural setting as well as the breadth of incentive designs tested contribute to the robustness of this finding. While it is not surprising that participant characteristics such as age, deprivation level and previous chlamydia history would affect the likelihood of returning a sample for testing, non-cash financial incentives, at least in some form, might be expected to alter any cost/benefit calculation regarding whether or not to return a sample. Even more so when well-known theoretical concepts from behavioral economics motivated the design of incentive schemes tested here.

That we find no effect is not to say that the theories behind them do not hold, rather that context matters – and this context does not support them. These results could be context specific to this largely Internet-based method of undertaking chlamydia screening and/or for STI screening in general, though other studies have shown some effects in precisely this context. The majority of those requesting a test kit may have some personal reason to do so and thus are not swayed by any extra enticement. The control group's return rate would be considered high for at home chlamydia testing (Ford, Viadro, & Miller, 2004; Sacks-Davis, Gold, Aitken, & Hellard, 2010), but is in line with Freetest.me's usual return rate. Curiously, about 30% of kits requested from Freetest.me are normally not returned, suggesting that even if young people have some reason or reasons to request one, these reasons are not strong enough to induce return in about one in three cases. The reasons why incentives cannot nudge this number downwards is worthy of further exploration.

This study's findings support the lack of effect found in Low et al. (2007) for a £10 voucher on chlamydia screening in the UK. Our findings differ from those of Zenner et al. (2012) who found a small but significant effect on chlamydia screening uptake among the same population group tested here when offering participants vouchers. Zenner et al. (2012) differs considerably in design to this study as it exploits differences in PCTs' uses of financial incentives for chlamydia screening and therefore could not control for how individual patient characteristics might influence the effect of financial incentives.

A number of studies have found financial incentives to be effective in chlamydia screening (Currie et al., 2010; Currie et al., 2013; Niza et al., 2013) as well as in other health contexts such as smoking cessation in pregnancy (Lumley et al., 2009), smoking more generally when using a large sample and incentive size (Volpp et al., 2009) and vaccinations (Seal et al., 2003; Topp et al., 2013). In Currie et al. (2010), the effect of the incentive could not be isolated from the fact that those given financial incentives were offered screening via text message versus a comparison group with no incentive receiving the screening offer via student organizations and media sources. The study examined two chlamydia screening program options rather than the effect of incentives in isolation. Currie et al. (2013) took place in a community pharmacy-based chlamydia screening program. They found a 93% return rate when young people returned the kit directly to the pharmacy where they picked it up and received an immediate reward of $A10 on specimen return (along with filling in a questionnaire and leaving contact details). Our study may present a barrier for young people by requiring that they mail back a specimen. The design of Niza et al. (2013) also allowed participants to receive the £5 voucher immediately upon kit return or be entered into the lottery. Niza et al. (2013) covered largely the same population (started at 18 rather than 16 years of age) but was a sample of London university students given the offer of a test kit and therefore might inherently differ from the England-wide population requesting test kits in this study. The cluster randomization design in Niza et al. (2013) also differs from the individual-level randomization undertaken in this study and thus could account for some of the heterogeneity in findings.

As for successful financial incentives programs in other health contexts, standard economic theory would suggest that if incentives were sufficiently large enough, they would evoke the expected behavioral response but this is an inefficient, impractical and financially unsustainable policy solution.

This study also demonstrates that non-cash financial incentives had no disproportionately greater or weaker effects depending on participant socioeconomic status as measured used IMD scores. Although we see that IMD score predicts specimen sample return rate and therefore deprivation level plays a role in screening uptake, deprivation does not diminish or increase the effectiveness of the variety of incentive structures. This finding offers empirical evidence that incentives may not be as effective as one might hope to reduce inequalities in the uptake of even short-term medical adherence behaviors (Oliver & Brown, 2012). The financial incentives literature has not extensively examined the interaction between socioeconomic status and financial incentives and warrants further attention to see if these results hold across context and population.

While IMD scores figure prominently in national and local policy decision-making in the UK, they have methodological limitations that must be recognized. When studying people and their behavior, this measure may be relatively weak as IMD scores capture area level information but our data are individual level. This may be a particular issue when conducting a study with young people because young people tend to live in areas of higher deprivation but not necessarily be as deprived as the area would reflect in their own household. IMD scores are also not a direct measure of deprivation in the same way that individual household income or unemployment would be. They provide a thorough picture of deprivation but at the area, not individual level (Department for Communities and Local Government, 2011). Even with these caveats, IMD is appropriate as an estimate for socioeconomic status given the policy implications for this research and our inability to collect direct measures of socioeconomic status from the sample population.

Another potential limitation of this study is the selective sample. The population consists of already motivated individuals, which may have implications for the extent to which external motivation in the form of non-cash financial incentives makes a difference in behavior. The sample is also slightly less deprived than the general population of England. The study also focuses on an Internet-based means of test requests, which may bias the sample as it is those who have access to and the ability to use a computer or a cell phone with mobile access. Given only the small difference in our mean sample IMD scores and those of England, this bias is only a limited concern. The intervention also targets young people for whom the Internet is part of their everyday life. Chlamydia testing also differs from other types of testing because the process requires returning the kit via the dispensing system for a result and does not provide an instant response such as in the case of, for example, pregnancy tests.

To our knowledge, this is the largest randomized experiment testing financial incentives of any kind in the context of STIs and one of the largest, most comprehensive trials of financial incentives to date. We also have data on a variety of relevant characteristics for our sample beyond demographic variables including sexual history and previous test results. This allows us to control for factors that may influence return rates beyond incentives and more readily isolate any effect of incentives.

In conclusion, our study suggests that non-cash financial incentives do not appear to affect the rate of specimen return in a chlamydia screening program where individuals request test kits online, receive them in the mail and return them by mail and that these effects are no stronger or weaker depending on socioeconomic status. Our study marks a step forward in the examination of incentives because of its experimental design in a natural setting, with a large sample size and testing of a variety of incentives. To examine some of the issues raised here further, a randomized experiment of specific population groups might be most useful for targeting the implementation of incentives to at-risk populations (e.g. those with a prior positive test, higher poverty levels). One might also consider involving the target population to design incentives tailored to them in efforts to improve on effectiveness. In the meantime, we need to be alert to the fact that incentives' effectiveness, just like any other intervention designed to influence behavior, will depend greatly on context (Dolan, Hallsworth, Halpern, Metcalfe, & Viaev, 2011).

## Figures and Tables

**Table 1:** Incentive types tested.

Group name	Round	Description	Incentive contingent upon returning the kit
Control	1 + 2	No incentive offer	N/A
**Voucher**			
GBP 5 voucher	1 + 2	Receive £5 Tesco voucher	Yes
GBP 10 voucher	2	Receive £10 Tesco voucher	Yes
**Lottery**			
Lottery with expected value (EV) of £5	1	Entered into a lottery with a 90% chance of £0 payoff and a 10% chance of a £50 Tesco voucher	yes
**Choice – certain reward versus lottery**			
Choice with EV of GBP 5	1	Given choice of receiving a £5 Tesco voucher or being entered into the lottery described above	yes
Choice with EV of GBP 10	2	Given choice of receiving a £10 Tesco voucher or being entered into a lottery with a 90% chance of £0 payoff and a 10% chance of a £100 Tesco voucher	Yes
**Endowment**			
GBP 5 endowment	1	Receive £5 Tesco voucher with kit	No
GBP 10 endowment	2	Receive £10 Tesco voucher with kit	No
**Charity**			
GBP 5 charity	1	Receive £5 donation on their behalf to Children in Need (*a UK children's charity*)	Yes

**Table 2:** Descriptive statistics of the sample by study round.

	Round 1 (*n* = 1489)	Round 2 (*n* = 1499)	Total (*n* = 2988)
	Mean	Mean	Mean
*Returned kit within 30 days*	71.3	70.8	71.0
*Incentive structure*			
Control	16.8	20.0	18.4
GBP 5 voucher	16.5	20.2	18.4
GBP 10 voucher	–	20.1	10.1
Lottery EV GBP 5	16.6	–	8.3
Choice EV GBP 5	16.6	–	8.3
Choice EV GBP 10	–	20.3	10.2
GBP 5 endowment	16.8	–	8.4
GBP 10 endowment	–	19.3	9.7
GBP 5 charity	16.7	–	8.3
*Gender*			
Male	32.6	34.7	33.7
*Level of poverty/deprivation*			
Index of Multiple Deprivation (IMD) Score^b^	20.4 (14.34)	20.0 (13.92)	20.2 (14.13)
*Age*			
16–19 years	33.0	28.4	30.7^a^
20–24 years	66.3	66.6	66.4
Age missing	0.7	5.0	2.9^a^
*Ethnicity*			
White	88.4	84.6	86.5^a^
Black	1.05	1.3	1.4
Asian	1.7	0.9	1.3
Other race	0.3	0.3	0.3
Mixed ethnicity	3.1	3.1	3.1
Unknown	4.9	9.7	7.3^a^
*New partner in the last 3 months*			
Yes	62.8	60.4	61.6
No	26.8	23.7	25.3
Unknown	10.4	15.9	13.2^a^
*Two or more sexual partners in last 12 months*			
Yes	68.2	63.0	65.6^a^
No	22.0	21.3	21.7
Unknown	9.80	15.6	12.7^a^
*Chlamydia test in the last 12 months*			
Yes, positive test	33.4	33.0	33.2
Yes, negative test	8.30	7.5	7.9
No	48.8	46.3	47.6
Unknown	9.50	13.2	11.4^a^
*Condom use when last had sex*			
Yes	16.9	16.3	16.6
No	71.5	67.9	69.7^a^
Unknown	11.6	15.7	13.7^a^

Notes: A response of ‘unknown’ means that the respondent was asked the question but responded ‘ I'd rather not say’ in the online questionnaire when requesting his/her kit or requested their test kit via text message and therefore did not have the opportunity to fill in the online questionnaire. ‘Age missing’ includes 8 participants who were either over the age of 24 or gave an inaccurate age figure (e.g. 100, 111). They would not have been eligible for NCSP testing and therefore were denoted as ‘age missing’ along with those who requested their kit by text and did not report an age.

a Statistically significant difference (at the 95% level) in means between characteristics of sample in Round 1 and Round 2.

b Figure in parenthesis is standard deviation.

**Table 3:** Baseline characteristics by group, Round 1. Figures are mean values (95% CI).

	Control (*n* = 250)	GBP 5 voucher (*n* = 246)	Lottery with EV of GBP 5 (*n* = 247)	Choice with EV of GBP 5 (*n* = 247)	GBP 5 endowment (*n* = 250)	GBP 5 charity (*n* = 249)
*Socio-demographic characteristics*						
Male	0.364 (0.304–0.424)	0.333 (0.274–0.393)	0.304 (0.246–0.361)	0.227 (0.174–0.279)	0.400 (0.339–0.461)	0.329 (0.271–0.388)
Index of Multiple Deprivation (IMD) Score	20.8 (19.0–22.7)	19.6 (17.9–21.3)	21.7 (19.8–23.5)	19.1 (17.3–20.9)	20.0 (18.3–21.7)	21.0 (19.1–22.8)
Age	20.5 (20.2–20.8)	20.8 (20.6–21.1)	20.7 (20.4–21.0)	20.4 (20.1–20.7)	20.5 (20.1–20.8)	20.8 (20.5–21.1)
White	0.852 (0.808–0.896)	0.886 (0.846–0.926)	0.883 (0.842–0.923)	0.891 (0.852–0.930)	0.876 (0.835–0.917)	0.920 (0.886–0.954)
Black	0.012 (−0.002–0.026)	0.020 (0.003–0.038)	0.016 (0.000–0.032)	0.008 (−0.003–0.019)	0.012 (−0.002–0.026)	0.020 (0.003–0.038)
Asian	0.032 (0.010–0.054)	0.012 (−0.002–0.026)	0.012 (−0.002–0.026)	0.016 (0.000–0.032)	0.028 (0.007–0.049)	0.004 (−0.004–0.012)
Mixed Ethnicity	0.040 (0.016–0.064)	0.024 (0.005–0.044)	0.036 (0.013–0.060)	0.024 (0.005–0.044)	0.040 (0.016–0.064)	0.020 (0.003–0.038)
*Sexual history*						
No new partner in the last 3 months	0.272 (0.216–0.328)	0.280 (0.224–0.337)	0.296 (0.238–0.353)	0.247 (0.193–0.301)	0.240 (0.187–0.293)	0.273 (0.217–0.329)
Not had two or more partners in last 12 months	0.232 (0.179–0.285)	0.236 (0.182–0.289)	0.223 (0.170–0.275)	0.206 (0.156–0.257)	0.188 (0.139–0.237)	0.237 (0.184–0.290)
Yes, positive chlamydia test in the last 12 months	0.304 (0.247–0.361)	0.313 (0.255–0.371)	0.372 (0.312–0.433)	0.328 (0.269–0.387)	0.320 (0.262–0.378)	0.365 (0.305–0.426)
Yes, negative chlamydia test in the last 12 months	0.064 (0.033–0.095)	0.089 (0.054–0.125)	0.093 (0.057–0.130)	0.089 (0.053–0.125)	0.092 (0.056–0.128)	0.068 (0.037–0.100)
No condom use when last had sex	0.696 (0.639–0.753)	0.760 (0.706–0.814)	0.688 (0.630–0.746)	0.709 (0.651–0.766)	0.744 (0.690–0.798)	0.691 (0.633–0.749)
Same sex partner in the last 3 months	0.032 (0.010–0.054)	0.008 (−0.003–0.019)	0.024 (0.005–0.044)	0.000 (0.000–0.000)	0.052 (0.024–0.080)	0.044 (0.018–0.070)
Partners of both sexes in the last 3 months	0.032 (0.010–0.054)	0.016 (0.000–0.032)	0.024 (0.005–0.044)	0.016 (0.000–0.032)	0.012 (−0.002–0.026)	0.032 (0.010–0.054)

**Table 4:** Baseline characteristics by group, Round 2. Figures are mean values (95% CI).

	Control (*n* = 299)	GBP 5 voucher (n-303)	GBP 10 voucher (n-302)	Choice with EV of GBP 10 (*n* = 305)	GBP 10 endowment (*n* = 290)
*Socio-demographic characteristics*					
Male	0.365 (0.310–0.419)	0.333 (0.280–0.387)	0.348 (0.294–0.402)	0.344 (0.291–0.398)	0.345 (0.290–0.400)
Index of Multiple Deprivation (IMD) Score	19.3 (17.7–20.8)	20.3 (18.8–21.9)	20.2 (18.9–21.7)	19.9 (18.3–21.4)	20.2 (18.6–21.9)
Age	20.7 (20.4–20.9)	20.7 (20.4–20.9)	20.8 (20.6–21.1)	20.8 (20.6–21.1)	20.8 (20.6–21.1)
White	0.836 (0.794–0.878)	0.851 (0.811–0.892)	0.844 (0.803–0.885)	0.836 (0.794–0.878)	0.862 (0.822–0.902)
Black	0.013 (0.000–0.026)	0.007 (−0.003–0.016)	0.017 (0.002–0.031)	0.020 (0.004–0.035)	0.010 (−0.001–0.022)
Asian	0.007 (−0.003–0.016)	0.013 (0.000–0.026)	0.010 (−0.001–0.021)	0.013 (0.000–0.026)	0.003 (−0.003–0.010)
Mixed Ethnicity	0.027 (0.008–0.045)	0.030 (0.010–0.049)	0.036 (0.015–0.058)	0.039 (0.017–0.061)	0.024 (0.006–0.042)
*Sexual history*					
No new partner in the last 3 months	0.244 (0.195–0.293	0.267 (0.217–0.317)	0.228 (0.181–0.276)	0.223 (0.176–0.270)	0.224 (0.176–0.272)
Not had two or more partners in last 12 months	0.191 (0.146–0.235)	0.224 (0.177–0.272)	0.225 (0.178–0.273)	0.213 (0.167–0.259)	0.214 (0.166–0.261)
Yes, positive chlamydia test in the last 12 months	0.351 (0.297–0.406	0.307 (0.255–0.359)	0.348 (0.294–0.402)	0.318 (0.265–0.371)	0.324 (0.270–0.378)
Yes, negative chlamydia test in the last 12 months	0.070 (0.041–0.099)	0.079 (0.049–0.110)	0.073 (0.043–0.102)	0.075 (0.046–0.105)	0.076 (0.045–0.107)
No condom use when last had sex	0.656 (0.601–0.710)	0.657 (0.603–0.711)	0.682 (0.629–0.735)	0.692 (0.640–0.744)	0.714 (0.661–0.766)
Same sex partner in the last 3 months	0.017 (0.002–0.031)	0.010 (−0.001–0.021)	0.013 (0.000–0.026)	0.013 (0.000–0.026)	0.014 (0.000–0.027)
Partners of both sexes in the last 3 months	0.027 (0.008–0.045)	0.017 (0.002–0.031)	0.026 (0.008–0.045)	0.020 (0.004–0.035)	0.024 (0.006–0.042)

**Table 5:** Predicting chlamydia sample return.

	Sample returned in 30 days from day of kit request *Round 1*		Sample returned in 30 days from day of kit request *Round 2*		Sample returned in 30 days from day of kit request *Round 1 + 2*	
	Odds ratio	*z*-stat	Odds ratio	*z*-stat	Odds ratio	*z*-stat
*Incentive structures*						
GBP 5 voucher	1.03	0.13	1.35	1.55	1.20	1.31
GBP 10 voucher	–	–	1.28	1.31	1.20	1.04
Lottery EV GBP 5	0.96	−0.21	–	–	1.06	0.30
Choice EV GBP 5	0.96	−0.19	–	–	1.03	0.15
Choice EV GBP 10	–	–	1.06	0.32	1.02	0.13
GBP 5 endowment	1.12	0.53	–	–	1.17	0.79
GBP 10 endowment	–	–	0.89	−0.63	0.87	−0.82
GBP 5 charity	0.88	−0.60	–	–	0.99	−0.06
*Socio-demographic characteristics*						
Male	1.03	0.21	0.83	−1.42	0.95	−0.64
IMD	0.99^a^	−3.23	0.99	−1.28	0.99^a^	−3.11
20–24 years	1.49^a^	3.16	1.22	1.41	1.35^a^	3.21
Black	0.88	−0.32	0.90	−0.21	0.84	−0.53
Asian	0.48^c^	−1.81	0.95	−0.09	0.61	−1.56
Mixed ethnicity	1.13	0.31	1.05	0.13	1.11	0.39
*Sexual history*						
No new partner in the last 3 months	0.87	−0.85	0.84	−1.04	0.87	−1.29
Not had two or more partners in last 12 months	1.32	1.61	0.74^c^	−1.74	1.00	0.00
Yes, positive chlamydia test in the last 12 months	1.54^a^	2.80	1.13	0.80	1.34^a^	2.56
Yes, negative chlamydia test in the last 12 months	1.25	1.02	0.74	−1.13	0.94	−0.34
No condom use when last had sex	0.68^b^	−2.16	1.40^c^	1.85	0.98	−0.21
Same sex partner in the last 3 months	0.47^b^	−2.02	1.07	0.12	0.62^c^	−1.66
Partners of both sexes in the last 3 months	0.43^b^	−2.23	0.16^a^	−4.77	0.27^a^	−5.05
*Study round 1*	–	–	–	–	0.93	−0.49
*Constant*	2.76^a^	3.91	2.43^a^	2.88	2.51^a^	4.40
Number of observations	1489		1499		2988	
Wald *χ*^2^ (prob > *χ*^2^)	82.71(0.000)		160.88 (0.000)		197.89 (0.000)	
Log pseudolikelihood	−852.2		−788.8		−1678.1	
Pseudo *r*^2^	0.046		0.129		0.067	

Note: Reference categories are control group, age 16–19 years, white, new partner in last 3 months, no Chlamydia test in the last 12 months, condom use when last had sex, different sex partner in last 3 months and Study Round 2. Specifications also control for Strategic Health Authority in which individuals live, a proxy for region of England and ‘unknown’ responses to all independent variables (‘no answers’ and text message requests therefore questions were not asked). All models clustered by first three letters of the postcode with varying numbers of clusters based on sample size.

a Significant at 1%.

b Significant at 5%.

c Significant at 10%.

**Table 6:** The impact of incentives according to socioeconomic status.

	Sample returned in 30 days from day of kit request *Round 1*		Sample returned in 30 days from day of kit request *Round 2*		Sample returned in 30 days from day of kit request *Round 1 + 2*	
	Odds ratio	*z*-stat	Odds ratio	*z*-stat	Odds ratio	*z*-stat
*Incentive structure*						
IMD*GBP 5 voucher	1.00	−0.07	0.99	−0.40	1.00	−0.14
IMD*GBP 10 voucher	–	–	1.01	0.64	1.01	1.26
IMD*lottery EV GBP 5	1.00	0.30	–	–	1.00	−0.15
IMD*choice EV GBP 5	1.01	0.44	–	–	1.00	0.02
IMD*choice EV GBP 10	–	–	0.98	−1.15	0.99	−0.78
IMD*GBP 5 endowment	0.99	−0.54	–	–	0.99	−1.11
IMD*GBP 10 endowment	–	–	0.99	−0.59	1.00	−0.24
IMD*GBP 5 charity	1.00	0.32	–	–	1.00	0.19
Number of observations	1489		1499		2988	
Wald *χ*^2^ (prob > *χ*^2^)	88.36 (0.000)		161.98 (0.000)		211.53 (0.000)	
Log pseudolikelihood	−851.5		−787.2		−1675.6	
Pseudo *r*^2^	0.047		0.131		0.069	

Note: These specifications include the same explanatory variables as specifications shown in Table 5. All models clustered by first three letters of the postcode with varying numbers of clusters based on sample size.

## Footnotes

1 Primary care trusts – purchase care for population in their geographic catchment area.
