# Supplementary material for: The effect of financial incentives on chlamydia testing rates: Evidence from a randomized experiment
Source: Soc Sci Med. 2014 Mar;105(100):140–8. doi: 10.1016/j.socscimed.2013.11.018 (PMC3969100; doi:10.1016/j.socscimed.2013.11.018)
Supplement: Supplementary file 1 [file mmc1.doc]

**Supplementary Appendix**

**Appendix 1. Slips in Freetest.me test kit, Round 1**

**Appendix 2. Slips in Freetest.me test kit, Round 2**

**
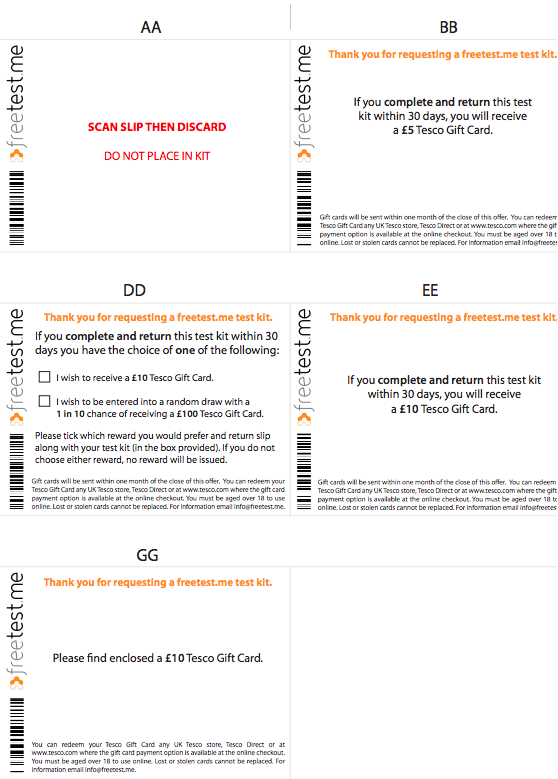
**

**Appendix 3. Flow of study participants, Round 1**

Lost to follow-up
(n= 0)

Randomized (n= 1500)

Randomized to
GBP 5 Endowment
(n= 250)

Randomized to
GBP 5 Charity
(n= 250)

Randomized to Choice Lottery EV GBP 5
(n= 250)

Randomized to Lottery EV GBP 5
(n= 250)

Randomized to
GBP 5 Voucher
(n= 250)

Randomized to Control
(n= 250)

Lost to follow-up (incorrectly scanned, label fell off test kit)
(n= 4)

Lost to follow-up (incorrectly scanned, label fell off test kit)
(n= 3)

Lost to follow-up (incorrectly scanned, label fell off test kit)
(n= 3)

Lost to follow-up
(n= 0)

Lost to follow-up (incorrectly scanned, label fell off test kit)
(n= 1)

Analyzed
(n= 250)

Analyzed
(n= 246)

Analyzed
(n= 247)

Analyzed
(n= 247)

Analyzed
(n= 250)

Analyzed
(n= 249)

**Appendix 4. Flow of study participants, Round 2**

Lost to follow-up
(n= 5)

Randomized (n= 1525)

Randomized to
GBP 10 Endowment
(n= 305)

Randomized to Choice Lottery EV GBP 10
(n= 305)

Randomized to
GBP 10 Voucher
(n= 305)

Randomized to
GBP 5 Voucher
(n= 305)

Randomized to Control
(n= 305)

Lost to follow-up (incorrectly scanned, label fell off test kit)
(n= 2)

Lost to follow-up (incorrectly scanned, label fell off test kit)
(n= 3)

Lost to follow-up (incorrectly scanned, label fell off test kit)
(n= 0)

Lost to follow-up
(n= 15)

Analyzed (n= 299)

Excluded from analysis because of no IMD score (n=1)

Analyzed (n= 303)

Analyzed (n= 302)

Analyzed (n= 305)

Analyzed (n= 290)

**Appendix 5. Freetest.me online questionnaire**

**
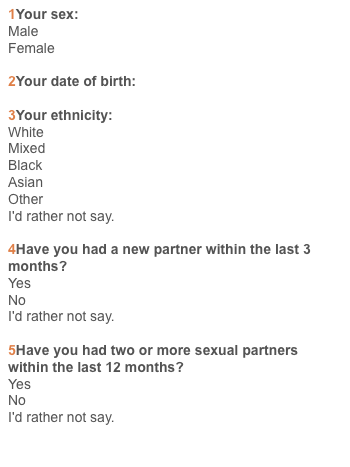
**

Note: Respondent only receives a test kit if responding ‘none of the above’ to question 9.
